# Short-term changes in ultrasound tomography measures of breast density and treatment-associated endocrine symptoms after tamoxifen therapy

**DOI:** 10.1038/s41523-023-00511-8

**Published:** 2023-03-15

**Authors:** Cody Ramin, Ruth M. Pfeiffer, Sharon Fan, Maeve Mullooly, Roni T. Falk, Kristine Jones, Neil E. Caporaso, Lisa Bey-Knight, Mark A. Sak, Michael S. Simon, David H. Gorski, Haythem Ali, Peter Littrup, Neb Duric, Mark E. Sherman, Gretchen L. Gierach

**Affiliations:** 1 Division of Cancer Epidemiology and Genetics, National Cancer Institute, Bethesda, MD USA; 2 School of Population Health, RCSI University of Medicine and Health Sciences, Dublin, Ireland; 3 Cancer Genomics Research Laboratory, Frederick National Laboratory for Cancer Research, Leidos Biomedical Research, Inc., Fredrick, MD USA; 4 Department of Oncology, Wayne State University, Barbara Ann Karmanos Cancer Institute, Detroit, MI USA; 5 Delphinus Medical Technologies, Novi, MI USA; 6 Michael and Marian Ilitch Department of Surgery, Wayne State University, Barbara Ann Karmanos Cancer Institute, Detroit, MI USA; 7 Henry Ford Cancer Institute, Henry Ford Health System, Detroit, MI USA; 8 Department of Imaging Sciences, University of Rochester, Rochester, NY USA; 9 Quantitative Health Sciences, Mayo Clinic, Jacksonville, FL USA

**Keywords:** Breast cancer, Cancer epidemiology

## Abstract

Although breast density decline with tamoxifen therapy is associated with greater therapeutic benefit, limited data suggest that endocrine symptoms may also be associated with improved breast cancer outcomes. However, it is unknown whether endocrine symptoms are associated with reductions in breast density after tamoxifen initiation. We evaluated treatment-associated endocrine symptoms and breast density change among 74 women prescribed tamoxifen in a 12-month longitudinal study. Treatment-associated endocrine symptoms and sound speed measures of breast density, assessed via novel whole breast ultrasound tomography (m/s), were ascertained before tamoxifen (T0) and at 1–3 (T1), 4–6 (T2), and 12 months (T3) after initiation. *CYP2D6* status was genotyped, and tamoxifen metabolites were measured at T3. Using multivariable linear regression, we estimated mean change in breast density by treatment-associated endocrine symptoms adjusting for age, race, menopausal status, body mass index, and baseline density. Significant breast density declines were observed in women with treatment-associated endocrine symptoms (mean change (95% confidence interval) at T1:−0.26 m/s (−2.17,1.65); T2:−2.12 m/s (−4.02,−0.22); T3:−3.73 m/s (−5.82,−1.63); *p*-trend = 0.004), but not among women without symptoms (*p*-trend = 0.18) (*p*-interaction = 0.02). Similar declines were observed with increasing symptom frequency (*p*-trends for no symptoms = 0.91; low/moderate symptoms = 0.03; high symptoms = 0.004). Density declines remained among women with detectable tamoxifen metabolites or intermediate/efficient *CYP2D6* metabolizer status. Emergent/worsening endocrine symptoms are associated with significant, early declines in breast density after tamoxifen initiation. Further studies are needed to assess whether these observations predict clinical outcomes. If confirmed, endocrine symptoms may be a proxy for tamoxifen response and useful for patients and providers to encourage adherence.

## Introduction

Tamoxifen, a selective-estrogen receptor modulator, is highly effective for breast cancer prevention among high-risk women^1–3^ and significantly reduces the risk of recurrence, second breast cancer, and mortality in the adjuvant setting among women diagnosed with estrogen receptor positive breast cancer^4–6^. Despite the therapeutic benefit of tamoxifen, adherence is low in the clinical setting with approximately 30–60% of women discontinuing tamoxifen before 5 years^7–11^. Prior studies have consistently found that treatment discontinuation is related, in part, to the development or worsening of vasomotor symptoms (e.g., hot flashes) and joint pain^11–15^. Therefore, identifying patients with treatment-related symptoms who are most likely to benefit from tamoxifen may focus attention on encouraging specific patients to persist with therapy and enable development of targeted studies that aim to ameliorate side effects.

Mammographic density decline after tamoxifen therapy is a proposed marker of treatment response^16,17^ as it has been associated with a lower risk of developing breast cancer in the chemopreventive setting^3^ and reduced risk of recurrence or death from breast cancer in the adjuvant setting^16,18–20^. Limited data further suggests that treatment-associated endocrine symptoms after tamoxifen initiation, including both vasomotor and joint symptoms related to modulation of the estrogen receptor, may be a potential indicator of more efficient metabolism of tamoxifen and therefore a predictor of favorable response^21–24^. However, whether treatment-associated endocrine symptoms are associated with reductions in breast density remains unknown.

For this study, we evaluated whether treatment-associated endocrine symptoms were associated with reductions in breast density among women undergoing tamoxifen therapy for clinical indication in the Ultrasound Study of Tamoxifen. We used a novel ultrasound tomography (UST) scanner to assess whole breast sound speed, a highly accurate and reliable estimate of volumetric breast density, repeatedly over time while avoiding ionizing radiation^25–27^. UST has received FDA approval for clinical use and was recently approved for breast cancer screening to enhance mammography among women with dense breasts^17,28^. We have previously shown rapid declines in density after tamoxifen initiation using UST scans and therefore UST may have utility to capture early responses to tamoxifen efficacy^17^. We further incorporated measures of circulating tamoxifen metabolites and sequenced *CYP2D6* to characterize tamoxifen metabolizer status. This 12-month longitudinal study provides a unique opportunity to examine relationships between serial measures of UST whole breast sound speed and repeated assessments of endocrine symptoms within the first year of tamoxifen initiation.

## Results

### Baseline characteristics

Participant characteristics are described in Table 1. At baseline, the mean age of participants was 51.4 years (SD = 9.0) and 66% of the women were premenopausal. Women who reported endocrine symptoms at baseline were slightly older, more likely to be Black women, and had higher body mass index (BMI) than those who did not report endocrine symptoms. Among those where *CYP2D6* metabolizer status was determined, 60% (*n* = 36) were efficient, 30% (*n* = 18) were intermediate, and 7% (*n* = 4) were poor metabolizers.

**Table 1:** Characteristics of women treated with tamoxifen in the Ultrasound Study of Tamoxifen.

Characteristics at baseline (T0)	Overall	Baseline endocrine symptoms^a^		
		No	Yes	*p* value^b^
N	74	28	46	
Age (years), mean (SD)	51.4 (9.0)	49.2 (10.4)	52.6 (7.8)	0.14
Race, *n* (%)				
Black	41 (55.4)	11 (39.3)	30 (65.2)	0.08
white	26 (35.1)	13 (46.4)	13 (28.3)	
other^c^	7 (9.5)	4 (14.3)	3 (6.5)	
Ethnicity, *n* (%)				
Hispanic/Latina	4 (5.4)	3 (10.7)	1 (2.2)	0.15
Non-Hispanic/Latina	70 (94.6)	25 (89.3)	45 (97.8)	
Education level, *n* (%)				
High school or less	23 (31.1)	10 (35.7)	13 (28.3)	0.79
Some college	24 (32.4)	8 (28.6)	16 (34.8)	
College or higher	27 (36.5)	10 (35.7)	17 (37.0)	
Body mass index (kg/m^2^), mean (SD)	30.3 (7.0)	28.7 (6.2)	31.3 (7.3)	0.10
Weight change (lbs.) at T3, mean (SD)^d^	0.67 (9.8)	1.6 (7.8)	0.2 (10.8)	0.52
Menopause status, *n* (%)				
Premenopausal	49 (66.2)	19 (67.9)	30 (65.2)	0.82
Postmenopausal	25 (33.8)	9 (32.1)	16 (34.8)	
Type of menopause, *n* (%)^e^				
Natural menopause	17 (68.0)	7 (77.8)	10 (62.5)	0.66
Surgical menopause	8 (32.0)	2 (22.2)	6 (37.5)	
Years since menopause, mean (SD)^e,f^	13.8 (9.3)	11.7 (7.1)	14.4 (10.5)	0.47
Pre- to postmenopausal by T3, *n* (%)^g^	4 (5.4)	0 (0.0)	4 (5.4)	0.29
Ever hormone replacement therapy, *n* (%)^e^	8 (32.0)	3 (33.3)	5 (31.3)	0.91
Antidepressant medication, *n* (%)^h^	12 (16.2)	2 (7.1)	10 (21.7)	0.11
Indication for tamoxifen therapy, *n* (%)				
High-risk	15 (20.3)	7 (25.0)	8 (17.4)	0.86
In situ	29 (39.2)	11 (39.3)	18 (39.1)	
Invasive with chemotherapy	7 (9.5)	2 (7.1)	5 (10.9)	
Invasive without chemotherapy	23 (31.1)	8 (28.6)	15 (32.6)	
Breast sound speed (m/s), median (25^th-^75^th^ percentile)	1449.2 (1444.0–1460.3)	1448.6 (1443.4–1459.4)	1449.7 (1444.4–1464.1)	0.54
Breast sound speed (m/s), tertiles, *n* (%)				
Tertile 1 (1434.4–1445.3)	24 (32.4)	9 (32.1)	15 (32.6)	0.96
Tertile 2 (1446.2–1456.6)	25 (33.8)	9 (32.1)	16 (34.8)	
Tertile 3 (1457.2–1499.6)	25 (33.8)	10 (35.7)	15 (32.6)	
Endocrine symptom frequency, *n* (%)				
None	28 (37.8)	28 (100)	0 (0.0)	-
Low/moderate	26 (35.1)	0 (0.0)	26 (56.5)	
High	20 (27.0)	0 (0.0)	20 (43.5)	
*CYP2D6* metabolizer status^i^, *n* (%)				
Poor	4 (6.7)	2 (8.3)	2 (5.6)	0.96
Intermediate	18 (30.0)	7 (29.2)	11 (30.6)	
Efficient	36 (60.0)	14 (58.3)	22 (61.1)	
Ultra	0 (0.0)	0 (0.0)	0 (0.0)	
Indeterminate	2 (3.3)	1 (4.2)	1 (2.8)	

Characteristics were ascertained at baseline unless otherwise specified.

^a^Endocrine symptoms were defined as vasomotor (i.e., hot flashes or flushes) and/or joint pain (i.e., stiffness or soreness in bone joints).

^b^*P* values were calculated using t-tests or Wilcoxon rank-sum tests for continuous variables and Fisher exact tests for categorical variables.

^c^Includes women with self-reported race as American Indian/Alaska Native (*n* = 1), Asian (*n* = 3), white & Black (*n* = 1), Black & American Indian/Alaska Native (*n* = 1), and missing (*n* = 1).

^d^Mean change from T0 to T3.

^e^Among postmenopausal women (*n* = 25).

^f^1 missing value for age at menopause.

^g^4 women changed from pre- to postmenopausal between T0 and T3.

^h^Among women with antidepressant use at baseline, 6 women reported use of medication that may inhibit *CYP2D6* (e.g., citalopram, paroxetine, fluoxetine, and bupropion).

^i^Among subgroup of women (*n* = 60).

### Trajectories of breast sound speed change by treatment-associated symptoms

**Fig. 1:**
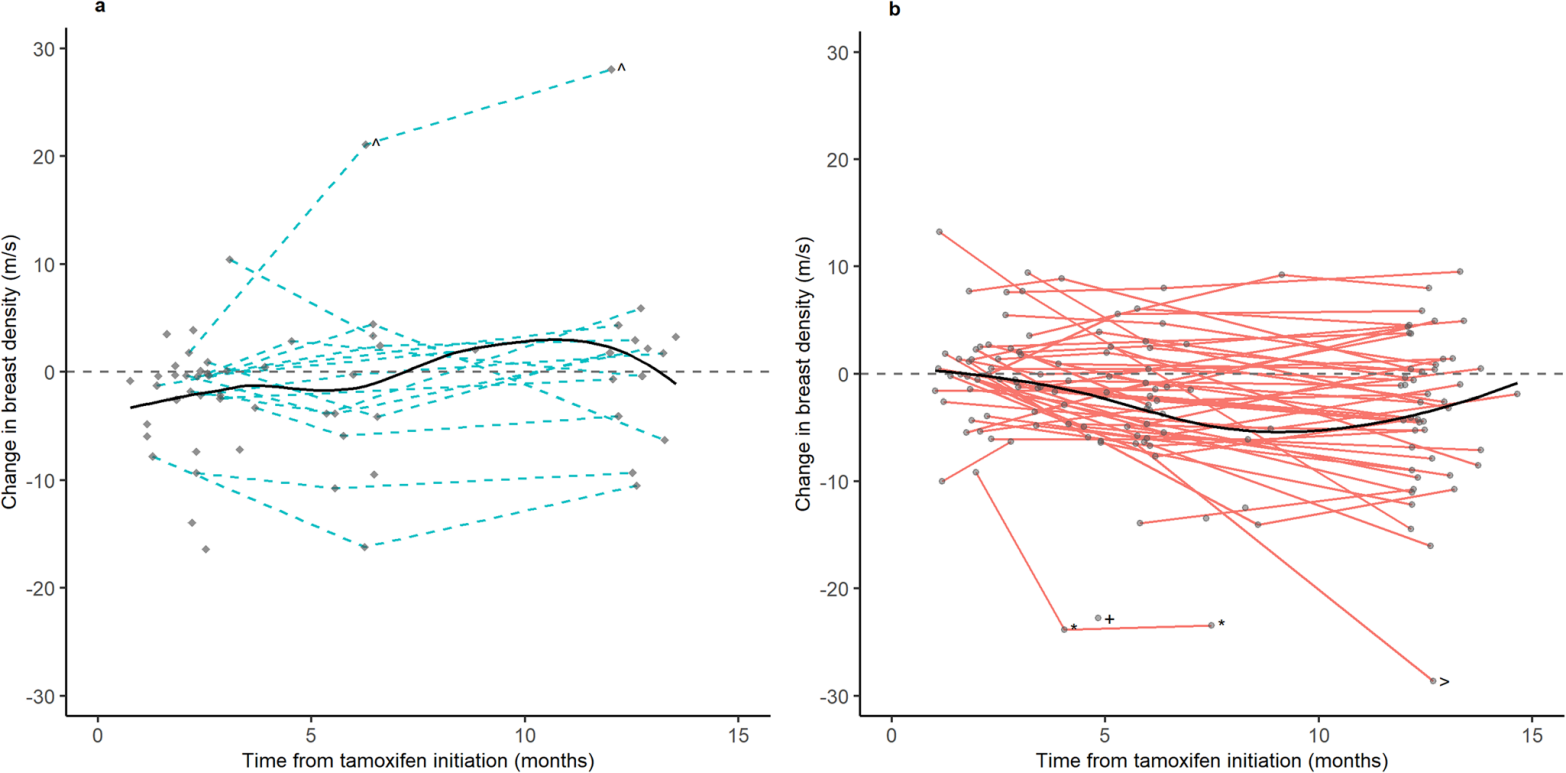
Trajectories of change in breast sound speed (m/s) by treatment-associated symptoms. **a** No treatment-associated symptoms. **b** Treatment-associated symptoms. Treatment-associated endocrine symptoms were defined as either emergent or worsening vasomotor symptoms and/or joint pain after tamoxifen initiation. Loess curve was fit between change in breast sound speed and time from tamoxifen initiation. Extreme values for change in breast sound speed (m/s) are indicated with symbols. Each symbol represents an observation from the same women. The caret represents a woman with an extreme value of breast sound speed increase and who reported weight loss from T0 to T2 of approximately 20 lbs. and T0 to T3 of 25 lbs. The plus, asterisk, and angle bracket symbols represent three separate women with an extreme value of breast sound speed decline and cessation of menstrual periods by T3.

Overall, women with treatment-associated endocrine symptoms had patterns of greater decline in sound speed compared to those who did not report treatment-associated symptoms after tamoxifen initiation (Fig. 1). Trajectories were similar when extreme values of sound speed change were excluded (see Supplementary Figure 1) and did not appear to largely differ for women with treatment-associated symptoms who reported high symptom frequency or low/moderate symptom frequency (see Supplementary Figure 2).

### Change in breast sound speed by treatment-associated endocrine symptoms

**Fig. 2:**
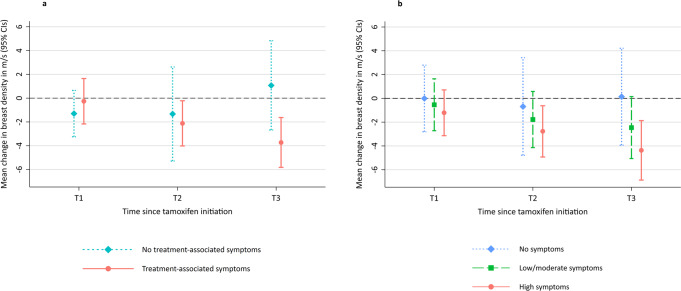
Multivariable-adjusted mean change in breast sound speed (m/s) by treatment-associated endocrine symptoms and endocrine symptom frequency. **a** By treatment-associated endocrine symptoms. Treatment-associated endocrine symptoms were defined as either emergent or worsening vasomotor symptoms and/or joint pain after tamoxifen initiation. P-trend for those with symptoms = 0.004, P-trend for those without symptoms = 0.18; P-interaction between endocrine symptom group and time = 0.02. **b** By endocrine symptom frequency. Endocrine symptom frequency was assessed with a Likert scale for symptom frequency per week (vasomotor and joint pain) and per day (vasomotor only). An endocrine symptom severity score was created by summing the values for each item and categorized into tertiles defined as no symptoms (score = 0), low/moderate (score = 1–5), and high (score=6+). P-trend for those with no symptoms = 0.91, low/moderate symptoms = 0.03, high symptoms = 0.004. P-interaction between endocrine symptom frequency and time = 0.61. All models were adjusted for age, race, menopause status, body mass index, and baseline sound speed. P-trends were calculated by modeling the time interval from tamoxifen initiation (T1, T2, T3) as a continuous variable using the Wald test. P-interactions were evaluated with multiplicative interaction terms using the Wald test. Error bars represent 95% confidence intervals.

In adjusted models, change in sound speed over time significantly differed by treatment-associated endocrine symptoms (*p*-interaction = 0.02) (Fig. 2a; see Supplementary Table 1). Statistically significant increasing declines in breast sound speed were observed among women with treatment-associated endocrine symptoms (mean change [95% CI] at T1: −0.26 m/s [−2.17, 1.65]; T2: −2.12 m/s [−4.02, −0.22]; T3: −3.73 m/s [−5.82, −1.63]; *p*-trend = 0.004), but not among women without symptoms (*p*-trend = 0.18). At the last UST scan, women who had experienced treatment-associated endocrine symptoms had a 5-fold higher odds of a decline of ≥2 m/s in sound speed compared to those without symptoms (OR = 5.23, 95% CI = 1.03–26.51) (see Supplementary Table 2).

### Change in breast sound speed by endocrine symptom frequency

Statistically significant declines in sound speed over time were greater in women with high symptom frequency (mean change [95% CI] at T1: −1.21 m/s [−3.14, 0.71]; T2: −2.77 m/s [−4.93, −0.62]; T3: −4.37 m/s [−6.87, −1.87]; *p*-trend = 0.004) than low/moderate symptom frequency (mean change [95% CI] at T1: −0.54 m/s [−2.72, 1.64]; T2: −1.78 m/s [−4.14, 0.58]; T3: −2.46 m/s [−5.06, 0.15]; *p*-trend = 0.03) (Fig. 2b, see Supplementary Table 3). No decline in sound speed was observed among women with no endocrine symptoms (*p*-trend = 0.91), but the test for interaction was not statistically significant (*p*-interaction = 0.61).

### Sensitivity and exploratory analyses

Results remained similar in analyses 1) restricted to premenopausal women (*n* = 49), 2) excluding women who discontinued tamoxifen or had undetectable levels of tamoxifen metabolites (*n* = 65; 9 women excluded), 3) adjusted for antidepressant use, or 4) adjusted for an indication for tamoxifen therapy (see Supplementary Tables 4–5). Among women with available *CYP2D6* status (*n* = 60), we found similar associations for change in breast sound speed by treatment-associated endocrine symptoms both before and after excluding the small number of women with poor *CYP2D6* metabolizer status (see Supplementary Table 4-5). As anticipated, poor or intermediate *CYP2D6* metabolizers had lower levels of endoxifen (median (range) for poor = 3.02 (2.29-4.16) ng/ml; intermediate = 6.03 (0.62-24.60) ng/ml) compared with efficient *CYP2D6* metabolizers (9.59 (0.25–32.4) ng/ml) (see Supplementary Table 6).

## Discussion

In this longitudinal study of patients prescribed tamoxifen for clinical indications, we found that women with treatment-associated endocrine symptoms after tamoxifen initiation experienced statistically significant declines in breast density within the first year of therapy, and this decline was stronger in women with more symptoms. Statistically significant declines in breast density were not observed among women without symptoms despite similar tamoxifen adherence. Associations remained among premenopausal women, a group in which tamoxifen is generally clinically indicated as first-line adjuvant endocrine therapy. These findings suggest that emergent/worsening symptoms after tamoxifen initiation may be a potential marker of treatment response.

To our knowledge, no prior study has examined treatment-associated endocrine symptoms and change in breast sound speed among women undergoing tamoxifen therapy. Previous studies have examined endocrine symptoms and breast cancer outcomes in women using tamoxifen therapy with positive results^21,22^. The Arimidex, Tamoxifen, Alone or in Combination (ATAC) trial found that postmenopausal breast cancer patients with endocrine symptoms at 3-months after tamoxifen initiation, including both vasomotor or joint symptoms, had a lower risk of breast cancer recurrence compared to those without symptoms^21^. Results were similar among women treated with aromatase inhibitors. Data from the Women’s Healthy Eating and Living (WHEL) randomized trial also indicated a lower risk of breast cancer recurrence among women treated with tamoxifen therapy who reported hot flashes than those who did not report hot flashes^22^. Additional studies examining both tamoxifen and aromatase inhibitors have also found that either vasomotor, musculoskeletal, or joint symptoms may be associated with improved breast cancer outcomes^23,24^ but results have been mixed^29,30^. Notably, prior studies on endocrine symptoms and improved breast cancer outcomes have been primarily based on clinical trial data and among postmenopausal women in the adjuvant setting. Together with the results from these trials, our findings suggest that treatment-associated endocrine symptoms may be a marker of treatment response. However, whether these symptoms and declines in breast density are associated with a lower risk of breast cancer in the prevention setting or recurrence, second breast cancer, and mortality in the adjuvant or neo-adjuvant setting, remains to be examined. An association between endocrine symptoms plus breast density, if confirmed, may have important clinical implications given that estrogen receptor-positive breast cancer incidence is rising^31^, particularly among younger women who are generally not eligible to take aromatase inhibitors unless they are at high-risk of recurrence, and in which situation, ovarian suppression or oophorectomy might be clinically indicated^32,33^.

The potential biological mechanisms underlying the association between endocrine symptoms and sound speed change are unclear. However, estrogen suppression plays an important pathophysiological role in the development of side effects, such as vasomotor and joint symptoms, and is related to declines in breast density. It is possible that women who do not efficiently metabolize tamoxifen have lower circulating levels of active tamoxifen metabolites^34^, are subsequently less likely to have endocrine symptoms^35–37^, and may have less decline in breast density^38^. In exploratory analyses, we found that women with poor or intermediate *CYP2D6* metabolizer status had lower levels of endoxifen compared with those with efficient *CYP2D6* status. Importantly, *CYP2D6* is responsible for the hydroxylation of N-desmethyltamoxifen to endoxifen, an active metabolite via which tamoxifen exerts the majority of its effects^39,40^. Although the distribution of *CYP2D6* metabolizer status was as expected in our study, limited sample size precluded a comprehensive evaluation of potential biological mechanisms. In addition, although density decline with tamoxifen therapy likely reflects decreases in stroma, it may also be influenced by other factors including BMI, weight change, and the menopausal transition^41–43^, which we accounted for in our analyses. Additional work is needed to understand the underlying mechanisms between tamoxifen metabolism, endocrine symptoms, and potential variation in breast density decline. Whether density decline is an intermediate endpoint and causally related to tamoxifen effectiveness or rather a marker of tamoxifen effectiveness still needs to be determined^16^.

Strengths of this study include longitudinal data on both self-reported endocrine symptoms and quantitative measures of whole breast UST sound speed within the first year of tamoxifen treatment. The use of nonionizing 3-D imaging with whole breast UST scans also provided the opportunity to assess the sound speed at short intervals during the first year of treatment in both high-risk women and breast cancer patients^27^. Our study further included information on both circulating tamoxifen metabolites, to reduce potential misclassification of tamoxifen therapy and as a proxy for tamoxifen adherence, and *CYP2D6* metabolizer status, to examine potential differences in tamoxifen metabolism. In addition, we were able to account for potential confounding by age, race, menopausal status, and BMI. Limitations of this study included the modest sample size, resulting in limited statistical power, particularly for stratified analyses. Further larger studies are needed to disaggregate results by race and ethnicity. In addition, repeated measures were only collected within the first year of tamoxifen treatment. However, prior studies have shown that the largest declines in breast density occur within the first 12–18 months of tamoxifen therapy^16,44^.

In conclusion, our study suggests that treatment-associated endocrine symptoms may be associated with declines in breast density and may be a marker of treatment response. Further studies are needed to confirm whether treatment-associated endocrine symptoms and breast density declines are associated with improved breast cancer outcomes. If treatment-associated symptoms are shown to be a surrogate for treatment response, this may be useful for patients and providers to improve and maintain tamoxifen adherence.

## Methods

### Study population

The Ultrasound Study of Tamoxifen enrolled 82 women prescribed tamoxifen for clinical indications and a comparison group of 165 women with screen-negative mammograms and no history of breast cancer at the Karmanos Cancer Institute (KCI) and Henry Ford Health Systems (HFHS) in Detroit, Michigan, USA between 2011–2014^25,27,45^. Women included in the study were aged 30–70 years, weighed ≤350 pounds, had breast size <22 cm in diameter, had no breast implants, reduction mammoplasty, or active skin infections/open chest wounds, and were not currently pregnant, breastfeeding, or taking endogenous hormones at study enrollment (i.e., oral contraceptives and menopausal hormone therapy)^17^. For the present analysis, we restricted our analytic population to the 82 women prescribed tamoxifen for clinical indications, which included women diagnosed with breast cancer after a routine screening (*n* = 31 in situ; n = 34 invasive) and high-risk patients referred for chemoprevention (based on personal breast cancer risk) (*n* = 17). Among these 82 women, we further excluded those who self-reported never starting tamoxifen (*n* = 6), those without a baseline UST scan (*n* = 1) and those without at least one follow-up UST scan (*n* = 1). Thus, 74 women were included in our final analytic population. All participants provided written informed consent and the study protocol was approved by the Institutional Review Boards at KCI, HFHS, and National Cancer Institute (NCI).

### Assessment of breast sound speed

**Fig. 3:**
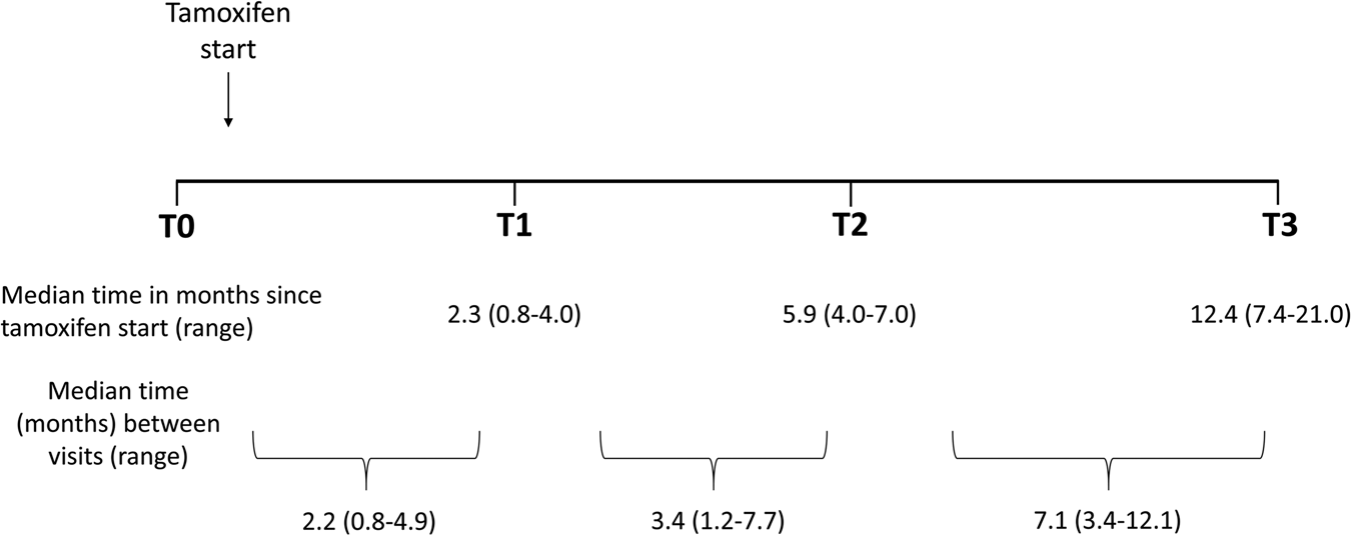
Study design for ultrasound tomography scans and endocrine symptom assessment in the Ultrasound Study of Tamoxifen. Ultrasound tomography scans and endocrine symptoms were ascertained prior to tamoxifen initiation (T0) and post-tamoxifen initiation (T1–T3).

Volume averaged sound speed (m/s), a surrogate measure of volumetric breast density^45,46^, was ascertained with whole breast UST scans conducted at baseline prior to tamoxifen initiation (T0) and approximately 1–3 months (T1), 4–6 months (T2), and 12-months (T3) after tamoxifen initiation (Fig. 3). Women with breast cancer had UST scans conducted on the contralateral unaffected breast. Methods to measure sound speed with UST scans have been previously described^17,25,27^. Briefly, all UST scans were conducted with an earlier prototype of the SoftVue system (manufactured by Delphinus Medical Technologies, Inc., Novi, MI, USA), which has been cleared by the FDA for clinical use and recently approved for breast cancer screening as an adjunct to mammography in women with dense breasts^25,27,28,45^. Volume average sound speed was estimated by averaging the sound speed of the breast across the tomographic DICOM image stacks (approximately 40–100 coronal image slices beginning from the chest wall to the nipple per UST scan). To assess change in sound speed between UST scans, we restricted image files to a common volume contained within all scans^25^. Sound speed was calculated from tomograms included in the common volume using automated scripts and then averaged together as a proxy for breast density^17^. UST sound speed has been shown to be a highly accurate and reliable estimate of volumetric breast density (intraclass correlation coefficient, ICC = 93.4%) and change in density (ICC = 70.4%)^25^, and may be more strongly associated with breast cancer risk than mammographic percent density^27^.

### Ascertainment of endocrine symptoms

Endocrine symptoms were self-reported at each UST scan prior to tamoxifen initiation (T0) and at approximately 1–3 months (T1), 4–6 months (T2), and 12 months (T3) post-tamoxifen initiation (Fig. 3). Symptoms were ascertained with a questionnaire administered by a research nurse (see Supplementary Methods for questionnaire details on endocrine symptoms). Endocrine symptoms included vasomotor and joint symptoms as these both relate to effects on endogenous estrogen levels^21^. Vasomotor symptoms were defined as hot flashes or flushes, and joint symptoms included stiffness or soreness in bone joints. Symptom frequency was defined as symptom occurrence in days per week and times per day (vasomotor symptoms only).

We examined endocrine symptoms with two approaches. Our primary approach was to examine treatment-associated endocrine symptoms which included emergent or worsening vasomotor and/or joint symptoms (yes, no). Endocrine symptoms were treated as time-dependent and updated at each UST scan. A woman was classified as having 1) emergent symptoms if she did not report any endocrine symptoms prior to that time or 2) increasing symptoms if she had reported them previously but symptoms had increased in frequency. Once a woman was classified as having either emergent or increasing symptoms, this classification was carried forward for the remainder of the analysis.

We additionally examined severity of endocrine symptoms based on how many times women experienced symptoms per day or week at each UST scan. We created a symptom frequency score by assigning a Likert scale with questions that assessed 1) how many days a woman experienced vasomotor and/or joint symptoms and 2) how many times per day a woman usually experienced hot flashes or flushes within the past two weeks. These responses were combined into the following scores for symptom occurrence: 0 = no symptoms; 1 = 1–5 days; 2 = 6–8 days; 3 = 9–13 days; 4 = every day, and summed together with the number of times women experienced hot flashes or flushes per day. The distribution of the resultant endocrine symptom frequency score was right-skewed, therefore the variable was categorized into tertiles of no symptoms (0), low-moderate symptoms (1–5), and high symptom frequency (6+).

### Genotyping and CYP2D6 phenotype assignment

#### DNA extraction

Saliva samples were collected from all participants at baseline using Oragene DISCOVER collection vials (DNAGenotek, Ontario, Canada) and received at ambient temperature. Source material aliquots of 850 uL were transferred into a new tube and aliquots were incubated at 50 °C for 60 min in a water bath. DNA was extracted using QIAsymphony DSP Virus/Pathogen Midi Kit on a QIAsymphony automated extraction instrument (Qiagen) following a customized version of the Complex800 CR2386 ID519 V2 protocol. Extracted DNA was quantified utilizing QuantiFluor dsDNA System (Promega Corporation, Madison, WI). Among our analytic population with saliva samples (*n* = 74), 60 participants had sufficient DNA that generated data for repeat amplification and sequencing.

#### CYP2D6 sequencing

*CYP2D6* was sequenced since tamoxifen is metabolized by the *CYP2D6* enzyme to 4-hydroxy-tamoxifen and endoxifen, and women with low enzyme activity may have less benefit from tamoxifen and be less likely to experience endocrine symptoms^22,37,47^. Sequencing was conducted at the NCI Cancer Genomics Research Laboratory, using a targeted long amplicon approach on the PacBio single molecule real-time (SMRT) sequencing platform. This approach facilitated accurate variant calling and direct haplotyping of the entire gene-locus, and produced reliable *CYP2D6* diplotypes. Methods were performed as described in the protocol from the manufacturer with some optimization to the PCR reactions and cycling parameters^48^.

A two-step PCR approach was used to generate amplicons with expected size of 6591 bp that spanned the *CYP2D6* gene. Amplicon coordinates in GRCh38 were chr22:42126037-42132625. During the first PCR, template-specific primers with universal priming site tags were used, where the template-specific primers span the *CYP2D6* gene. Primer sequences for the first PCR were /5AmMC6/GCAGTCGAACATGTAGCTGACTCAGGTCAC | ATGGCAGCTGCCATACAATCCACCTG (forward) and /5AmMC6/TGGATCACTTGTGCAAGCATCACATCGTAG | CGACTGAGCCCTGGGAGGTAGGTAG (reverse). During the second PCR, the first portion of each primer was used as a universal priming site to build in barcode sequences to allow for pooling after amplification. The template-specific portion of each primer was obtained from the research article by Buermans and colleagues^49^. Primers were ordered from Integrated DNA Technologies (IDT) and high-performance liquid chromatography (HPLC) purified.

The first PCR reaction included the following: 2ul(5 ng/ul) genomic DNA, 15 ul water, 2.5ul LA PCR Buffer II, 4ul 2.5 mM dNTPs, 0.5 ul each of 100uM primer(F/R), and 0.5ul Takara LA Taq DNA Polymerase HS. The cycling conditions were 1 cycle of 94 °C for 1 min, followed by 30 cycles of 98 °C for 10 seconds and 68 °C for 6 min, followed by a final extension at 72 °C for 10 min, and a hold at 4 °C. The second PCR utilized Barcoded Universal Primers (purchased from PacBio), which primed the universal priming site incorporated during the first PCR for amplification, and incorporated a unique barcode sequence for each sample to allow for pooling after amplification. The second PCR reaction included the following: 2ul first round PCR product (normalized to 1 ng/ul), 14 ul water, 2.5ul LA Taq PCR Buffer II, 4ul 2.5 mM dNTPs, 2ul each of 2uM Barcoded Universal Primers, and 0.5 ul Takara LA Taq DNA Polymerase HS. The cycling conditions were identical to the first round of PCR with the exception that only 10 cycles of annealing and extension were performed, instead of 30. All samples were pooled for SMRTbell library prep and sequencing on a single SMRT Cell 1 M on the PacBio Sequel instrument, according to the PacBio Protocol “Preparing SMRTbell Libraries Using Barcoded Universal Primers for Multiplexing Amplicons”, version 2^48^.

Circular Consensus Sequence (CCS) reads were generated using Circular Consensus Sequencing, demultiplexed using Lima, and mapped to GRCh38 using pbmm2. These steps were performed in SMRT Link 8.0. Mapped bams were inputted to Google Deep Variant and GATK Haplotype Caller, and variants were called. Variants that were called by both callers, and fell within the targeted amplicon region, were inputted to WhatsHap to phase variants by allele. An internal tool was developed to compare the variant call format (VCF) generated by WhatsHap to the PharmGKB *CYP2D6* Allele Definition Table^34,50^ and to assign a diplotype. Additional manual review of the WhatsHap VCF identified some instances of copy number changes based on read depth ratios of heterozygous variants. Diplotypes that could not be called based on the results of the assay included copy number changes with only one type of allele (no heterozygous variants), as well as some types of hybrids, since our primers were anchored in *CYP2D6. CYP2D6/CYP2D7* hybrids, which did not contain both of our primer sequences were not amplified and observed in the assay.

#### CYP2D6 phenotype

A *CYP2D6* activity score was assigned to each allele and summed for an overall activity score^34,51^. Corresponding phenotypes for the diplotypes were ascertained from the PharmGKB *CYP2D6* Diplotype-Phenotype Table^34,50^. *CYP2D6* phenotypes were classified as poor (activity score: 0), intermediate (activity score: >0–1.0), efficient (activity score: >1.0–2.0), or ultra-metabolizers (activity score: >2.0)^34,50^.

### Tamoxifen metabolites

Whole blood samples were collected 12 months after tamoxifen initiation. Tamoxifen metabolites were quantified in serum samples using liquid chromatography-tandem mass spectrometry (LC-MS/MS) at the Illinois Institute of Technology Research Institute^52,53^. Metabolites included: (Z)-tamoxifen, (Z)-n-desmethyl-tamoxifen, (Z)−4-OH-tamoxifen, and (Z)-endoxifen. Coefficients of variation (CVs) were <5% for all metabolites measured except for (Z)−4-OH-tamoxifen which was below the lower limit of detection in 7 participants and had a CV of 21%.

### Covariates

Information on demographics (e.g., age, race/ethnicity, education level), reproductive factors (e.g., menopausal status, type of menopause, date of menopause, menopausal hormone therapy) and regular medication use (e.g., antidepressants) were collected at each UST scan with questionnaires administered by the research nurse. Race (white, Black, other) and ethnicity (Hispanic/Latina, Non-Hispanic/Latina) were self-reported by the participants. Information on medication use included type, dose, frequency, duration, and indication. Regular medication use was defined as taken regularly at least 2 times per week in the last month. Height was measured at baseline and weight was ascertained at each scan to calculate BMI in kg/m^2^.

### Statistical analysis

Baseline characteristics were described overall and compared by baseline endocrine symptom status using t-tests or Wilcoxon rank-sum tests for continuous variables and Fisher exact tests for categorical variables. Absolute mean change in sound speed was calculated at each UST scan as the difference from baseline (T0). The distribution of mean change in sound speed was approximately normally distributed. Trajectories of sound speed change were examined by treatment-associated endocrine symptoms. We used linear regression to examine the mean change in sound speed over time by endocrine symptoms. Variances were estimated using generalized estimating equations to account for within-subject correlations over time^54^. Multivariable models were adjusted for a priori specified covariates including baseline age (continuous), race (white, Black/other), baseline menopausal status (premenopausal, postmenopausal), BMI (continuous), and sound speed prior to tamoxifen initiation (tertiles). BMI was treated as a time-varying variable in all models since it was a strong confounder. As change in menopausal status may have also been a strong confounder, in sensitivity analyses we excluded 4 women who changed from pre- to postmenopausal during follow-up (surgical or natural defined as ≥12 months since last menstrual period); however, this did not alter our results and therefore these women were retained in our analyses. We tested for linear trend by modeling the time interval from tamoxifen initiation (T1, T2, T3) as a continuous variable using the Wald test. To determine if sound speed change over time differed by endocrine symptoms, multiplicative interaction terms were evaluated using a Wald test. In a *post-hoc* analysis, we used logistic regression to estimate odds ratios (ORs) for the association between treatment-associated endocrine symptoms at each UST scan and a predetermined decline of ≥2 m/s in sound speed at the last UST scan (T3 minus T0). A decline of ≥2 m/s in sound speed was selected as a potentially clinically meaningful cut point based on results from our primary analysis and prior study^17^.

We conducted additional sensitivity analyses including 1) restricting analyses to premenopausal women (*n* = 49), 2) excluding women who discontinued tamoxifen or had undetectable levels of tamoxifen metabolites (as a proxy for tamoxifen adherence) (*n* = 9), 3) adjusting for antidepressant use since it may alter tamoxifen metabolism^55^, and 4) adjusting for tamoxifen indication (high-risk, in situ breast cancer, invasive breast cancer with/without chemotherapy).

In the subgroup with *CYP2D6* status (*n* = 60), we further examined change in sound speed over time by endocrine symptoms excluding women with poor *CYP2D6* metabolizer status (*n* = 4). In exploratory analyses, we also examined the distribution of tamoxifen metabolites by *CYP2D6* status, with particular emphasis on Z-endoxifen, the most potent metabolite synthesized by *CYP2D6*.

Analyses were conducted using SAS (version 9.4). Figures were made in R (version 4.2.0, Boston, MA) with the ggplot package and loess smoother, and Stata (version 16.0, College Station, TX). Two-sided *p*-values <0.05 were considered statistically significant.

### Reporting summary

Further information on research design is available in the Nature Research Reporting Summary linked to this article.

## Supplementary information

Supplemental Material

Reporting Summary

## Data Availability

The data underlying this article will be shared on reasonable request to the Principal Investigator of the Ultrasound Study of Tamoxifen (GLG). Phenotype and sequencing data used in this study have been deposited in the NCBI database of Genotypes and Phenotypes (dbGaP) and the Sequence Read Archive (SRA) with the primary accession code phs003183.v1.p1.

## Code availability

Access to analytic code used in this study is available upon reasonable request to the corresponding author.
