# Supplementary material for: Short-term changes in ultrasound tomography measures of breast density and treatment-associated endocrine symptoms after tamoxifen therapy
Source: NPJ Breast Cancer. 2023 Mar 15;9:12. doi: 10.1038/s41523-023-00511-8 (PMC10017770; doi:10.1038/s41523-023-00511-8)
Supplement: Supplementary file 1 — Supplemental Material [file 41523_2023_511_MOESM1_ESM.pdf]

Supplementary Materials

Supplementary Methods

Supplementary Figure 1. Trajectories for change in breast sound speed (m/s) by treatment-associated symptoms, excluding extreme values of sound speed change

Supplementary Figure 2. Trajectories for change in breast sound speed (m/s) by treatment-associated symptoms, including endocrine symptom frequency

Supplementary Table 1. Mean change in breast sound speed (m/s) by treatment-associated endocrine symptoms after tamoxifen initiation

Supplementary Table 2. Odds ratios and 95% CIs for treatment-associated endocrine symptoms at T1-T3 and ≥2 m/s decline in breast sound speed at T3

Supplementary Table 3. Mean change in breast sound speed (m/s) by endocrine symptom frequency after tamoxifen initiation

Supplementary Table 4. Sensitivity analyses for mean change in breast sound speed (m/s) by treatment-associated endocrine symptoms after tamoxifen initiation

Supplementary Table 5. Sensitivity analyses for mean change in breast sound speed (m/s) by endocrine symptom frequency after tamoxifen initiation

Supplementary Table 6. Distribution of tamoxifen metabolites by *CYP2D6* metabolizer status

Supplementary Methods

Ascertainment of endocrine symptoms

To capture vasomotor symptoms, participants were asked the following questions on both the baseline and follow-up questionnaires:

1. During the past 2 weeks, have you experienced hot flashes or flushes?

   1☐ YES

   2☐ NO

   1a. On how many days have you experienced hot flashes or flushes in the past 2 weeks?

   1☐ 1-5 days,

   2☐ 6-8 days,

   3☐ 9-13 days, or

   4☐ Every day?

   1b. On the days that you have hot flashes or flushes, how many times each day do you usually have them? If you are not sure, please provide your best guess.

   ☐☐☐ number of times per day

   1c. How much are you usually bothered by hot flashes or flushes?

   1☐ Not at all,

   2☐ Very little,

   3☐ Moderately, or

   4☐ A lot?

To identify joint pain, participants were asked the following questions on both the baseline and follow-up questionnaires:

1. During the past 2 weeks, have you experienced stiffness or soreness in your bone joints?

   1☐ YES

   2☐ NO

1a. On how many days have you experienced stiffness or soreness in your bone joints in the past 2 weeks? If you are not sure, please provide your best guess.

   1☐ 1-5 days,

2☐ 6-8 days,

3☐ 9-13 days, or

4☐ Every day?

1b. How much are you usually bothered by stiffness or soreness in joints?

1☐ Not at all,

2☐ Very little,

3☐ Moderately, or

4☐ A lot

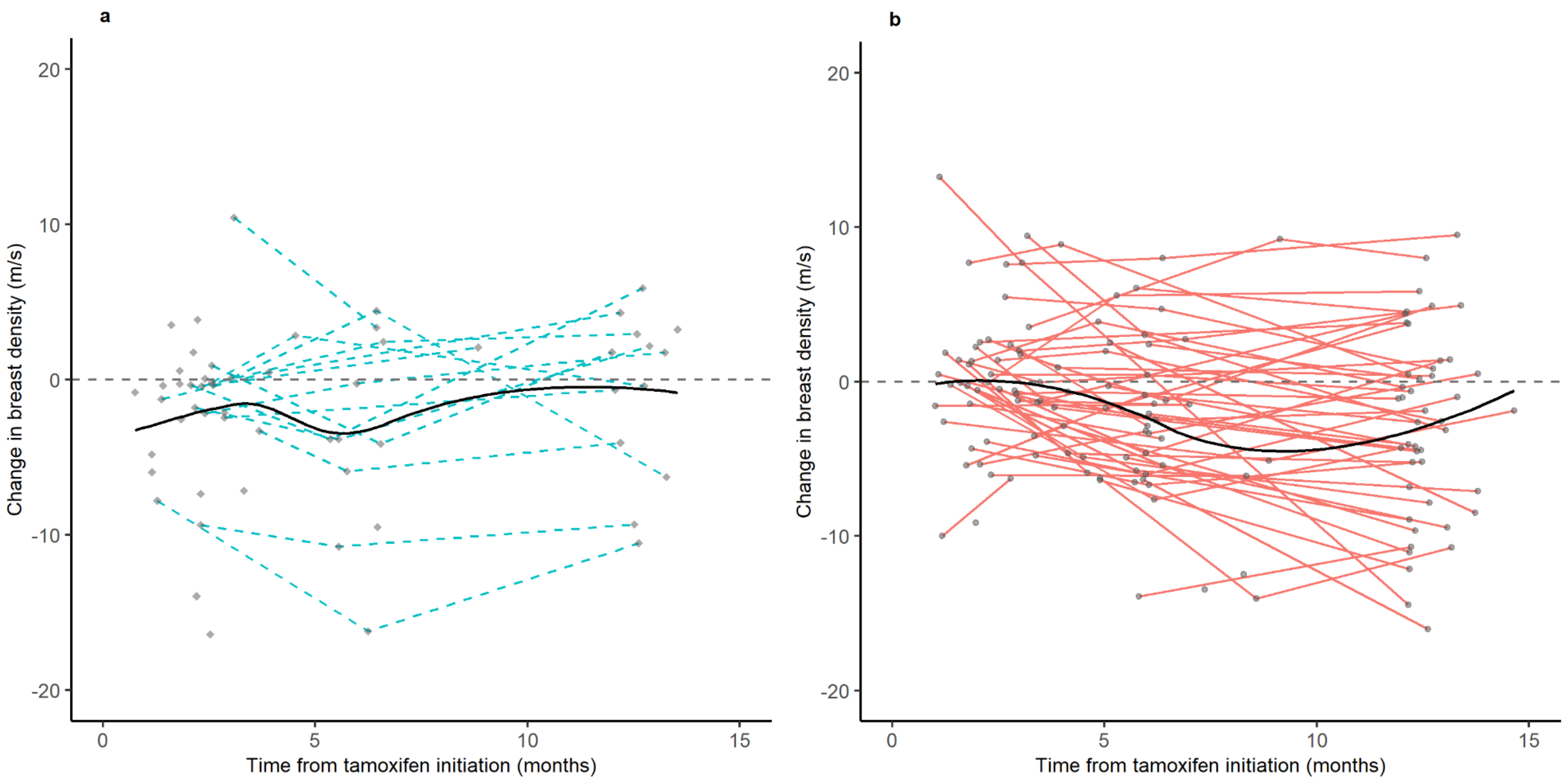

Supplementary Figure 1. Trajectories for change in breast sound speed (m/s) by treatment-associated symptoms, excluding extreme values of sound speed change

a. No treatment-associated symptoms. **b.** Treatment-associated symptoms. Treatment associated endocrine symptoms were defined as either emergent or worsening vasomotor symptoms and/or joint pain after tamoxifen initiation. Loess curve was fit between change in breast sound speed and time from tamoxifen initiation.

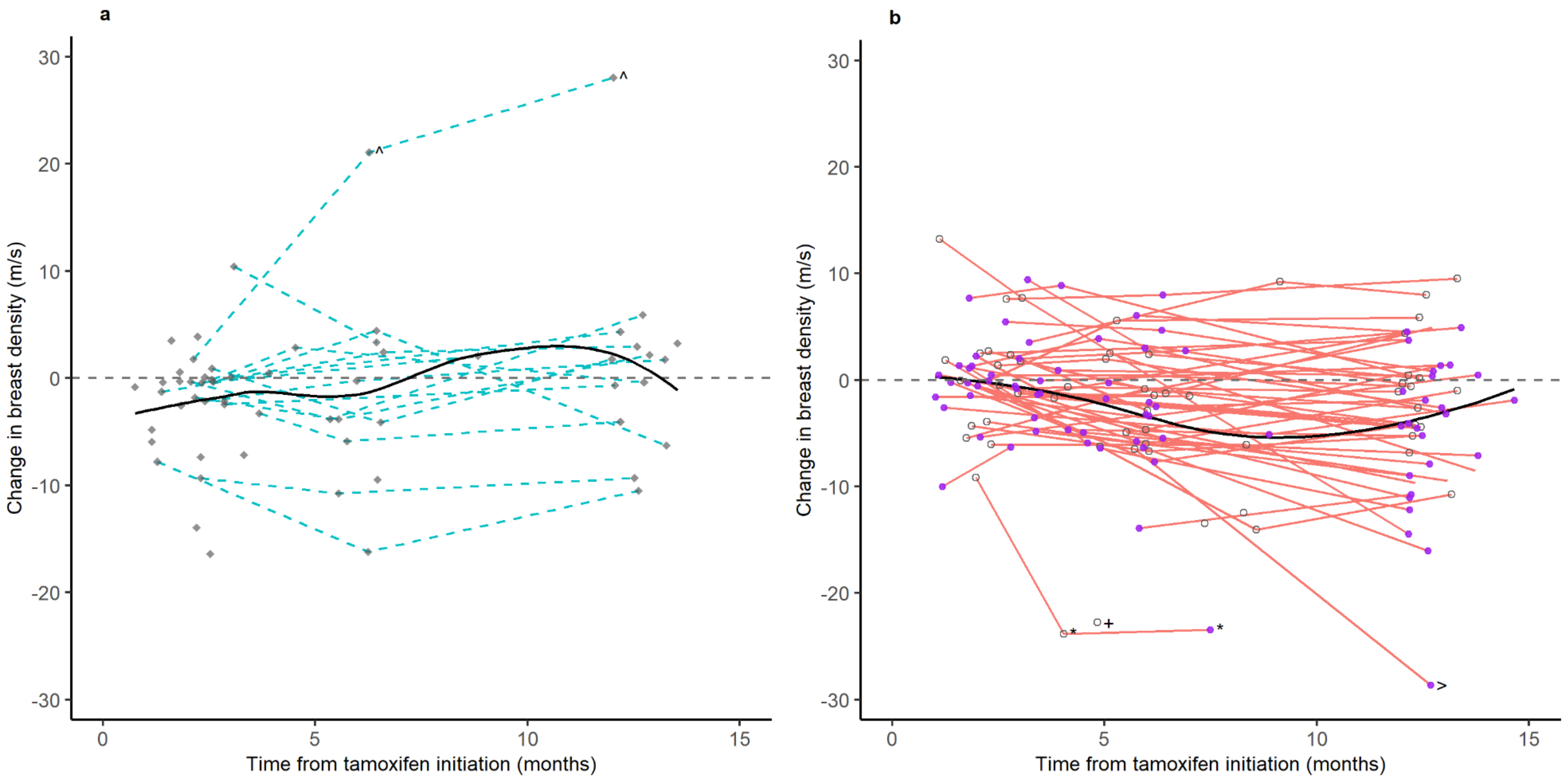

Supplementary Figure 2. Trajectories for change in breast sound speed (m/s) by treatment-associated symptoms, including endocrine symptom frequency

a. No treatment-associated symptoms. **b.** Treatment-associated symptoms. Treatment associated endocrine symptoms were defined as either emergent or worsening vasomotor symptoms and/or joint pain after tamoxifen initiation. Loess curve was fit between change in breast sound speed and time from tamoxifen initiation. Low/moderate symptom frequency is indicated with an unfilled circle and high symptom frequency is indicated with a purple filled circle. Among these women, 54% reported high symptom frequency during follow-up (63% at T1, 50% at T2, and 50% at T3). Extreme values for change in breast sound speed (m/s) are indicated with symbols. Each symbol represents an observation from the same women. The caret represents a woman with an extreme value of breast sound speed increase and who reported weight loss from T0 to T2 of

approximately 20 lbs. and T0 to T3 of 25 lbs. The plus symbol represents a woman with an extreme value of breast sound speed decline and who reported cessation of menstrual periods by T3. The asterisk represents a woman with an extreme value of breast sound speed decline and who reported cessation of menstrual periods by T3. The angle bracket represents a woman with an extreme value of breast sound speed decline and who reported cessation of menstrual periods by T3.

Supplementary Table 1. Mean change in breast sound speed (m/s) by treatment-associated endocrine symptoms after tamoxifen initiation

		Age-Adjusted[a]	Multivariable-Adjusted[b]
Time since tamoxifen initiation	No. of observations	Mean (95% CI)	Mean (95% CI)
No treatment-associated symptoms			
T1	31	-1.88 (-3.51 -0.25)	-1.30 (-3.25, 0.65)
T2	13	-1.97 (-5.85, 1.92)	-1.33 (-5.29, 2.62)
T3	15	0.56 (-3.09, 4.21)	1.07 (-2.68, 4.83)
p-trend[c]		0.14	0.18
Treatment-associated symptoms			
T1	48	-0.52 (-1.93, 0.90)	-0.26 (-2.17, 1.65)
T2	42	-2.56 (-4.13, -0.99)	-2.12 (-4.02, -0.22)
T3	58	-4.10 (-6.05, -2.15)	-3.73 (-5.82, -1.63)
p-trend[c]		0.002	0.004
p-interaction[d]		0.02	0.02

Treatment-associated endocrine symptoms were defined as either emergent or increasing vasomotor symptoms and/or joint pain after tamoxifen initiation

[a] Model adjusted for age (continuous)

[b] Model adjusted for age (continuous), race (white, Black/other), menopause status (premenopausal, postmenopausal), body mass index (continuous), baseline sound speed (m/s) (ordinal tertiles)

[c] P-trend was calculated by modeling time since tamoxifen initiation (T1-T3) as a continuous variable using the Wald test

[d] P-value for a Wald test of the interaction between endocrine symptom group and time since tamoxifen initiation

Supplementary Table 2. Odds ratios and 95% CIs for treatment-associated endocrine symptoms at T1-T3[a] and ≥2 m/s decline in breast sound speed at T3[b]

Endocrine symptoms	Events/ No. of women	OR (95% CI)[c]
T1 (N=68)		
No treatment-associated symptoms	15/30	1.00 (ref)
Treatment-associated symptoms	18/38	1.57 (0.48-5.09)
T2 (N=65)		
No treatment-associated symptoms	5/13	1.00 (ref)
Treatment-associated symptoms	28/52	4.25 (0.73-24.60)
T3 (N=73)		
No treatment-associated symptoms	4/14	1.00 (ref)
Treatment-associated symptoms	32/59	5.23 (1.03-26.51)

[a] Treatment associated endocrine symptoms were defined as either emergent or increasing vasomotor symptoms and/or joint pain after tamoxifen initiation at T1 to T3. Once classified as having either emergent or worsening symptoms, this classification was carried forward for the remainder of the analysis
[b] A decline of ≥2 m/s in breast sound speed was ascertained from change in sound speed from T3 minus T0
[c] Model adjusted for age (continuous), race (white, Black/other), menopause status (premenopausal, postmenopausal), body mass index (continuous), baseline sound speed (m/s) (ordinal tertiles)

Supplementary Table 3. Mean change in breast sound speed (m/s) by endocrine symptom frequency after tamoxifen initiation

		Age-Adjusted[a]	Multivariable-Adjusted[b]
Time since tamoxifen initiation	No. of observations	Mean (95% CI)	Mean (95% CI)
No symptoms			
T1	16	-0.58 (-2.95, 1.79)	-0.01 (-2.81, 2.79)
T2	9	-1.07 (-5.06, 2.93)	-0.69 (-4.79, 3.42)
T3	14	-0.24 (-4.29, 3.80)	0.14 (-3.94, 4.21)
p-trend[c]		0.53	0.91
Low/moderate			
T1	28	-0.87 (-2.89, 1.14)	-0.54 (-2.72, 1.64)
T2	24	-2.22 (-4.49, 0.05)	-1.78 (-4.14, 0.58)
T3	28	-2.65 (-4.93, -0.36)	-2.46 (-5.06, 0.15)
p-trend[c]		0.07	0.03
High			
T1	35	-1.47 (-2.86, -0.08)	-1.21 (-3.14, 0.71)
T2	22	-3.23 (-5.00, -1.45)	-2.77 (-4.93, -0.62)
T3	31	-4.89 (-7.49, -2.29)	-4.37 (-6.87, -1.87)
p-trend[c]		0.002	0.004
p-interaction[d]		0.55	0.61

Endocrine symptom frequency was assessed with a Likert scale for symptom frequency per week (vasomotor and joint pain) and per day (vasomotor only). An endocrine symptom frequency score was created by summing the values for each item and categorized into three groups defined as no symptoms (score=0), low/moderate (score=1-5), and high (score=6+)

[a] Model adjusted for age (continuous)

[b] Model adjusted for age (continuous), race (white, Black/other), menopause status (premenopausal, postmenopausal), body mass index (continuous), baseline sound speed (m/s) (ordinal tertiles)

[c] P-trend was calculated by modeling the time since tamoxifen initiation (T1-T3) as a continuous variable using the Wald test

[d] P-value for a Wald test of the-interaction between endocrine symptom score and time since tamoxifen initiation

Supplementary Table 4. Sensitivity analyses for mean change (95% CI)[a] in breast sound speed (m/s) by treatment-associated endocrine symptoms after tamoxifen initiation

	Restricted to premenopausal women	Excluded women who discontinued tamoxifen or had undetectable levels of tamoxifen metabolites	Adjusted for antidepressant use	Adjusted for case-type	Available *CYP2D6* status[b]	Excluded poor *CYP2D6* metabolizers[b]
Time since tamoxifen initiation						
No treatment-associated symptoms						
T1	-2.04 (-4.56, 0.49)	-1.32 (-3.33, 0.69)	-1.14 (-3.22, 0.93)	-2.44 (-4.20, -0.68)	-0.97 (-3.18, 1.24)	-1.19 (-3.56, 1.19)
T2	-0.21 (-5.59, 5.16)	-0.63 (-4.57, 3.31)	-1.17 (-5.14, 2.80)	-2.47 (-6.08, 1.13)	-0.52 (-5.02, 3.99)	-0.83 (-6.24, 4.57)
T3	0.31 (-4.82, 5.44)	1.69 (-2.35, 5.73)	1.24 (-2.54, 5.02)	0.01 (-3.22, 3.24)	1.35 (-2.80, 5.49)	1.36 (-3.22, 5.95)
p-trend[c]	0.41	0.34	0.18	0.19	0.20	0.16
Treatment-associated symptoms						
T1	0.04 (-2.19, 2.26)	-0.66 (-2.70, 1.39)	-0.13 (-2.27, 2.00)	-1.32 (-2.92, 0.28)	0.18 (-2.01, 2.38)	0.60 (-1.61, 2.81)
T2	-3.51 (-5.63, -1.39)	-2.66 (-4.63, -0.69)	-1.97 (-4.04, 0.09)	-3.22 (-5.00, -1.44)	-1.28 (-3.51, 0.94)	-1.16 (-3.53, 1.20)
T3	-5.02 (-7.47, -2.57)	-4.87 (-6.99, -2.75)	-3.60 (-5.66, -1.54)	-4.86 (-6.80, -2.91)	-3.76 (-6.13, -1.40)	-3.57 (-6.01, -1.14)
p-trend[c]	0.004	0.003	0.004	0.003	0.007	0.005
p-interaction[d]	0.05	0.01	0.02	0.02	0.02	0.02

Treatment-associated endocrine symptoms were defined as either emergent or increasing vasomotor symptoms and/or joint pain after tamoxifen initiation

[a] Model adjusted for age (continuous), race (white, Black/other), menopause status (premenopausal, postmenopausal), body mass index (continuous), baseline sound speed (m/s) (ordinal tertiles)

[b] Among subgroup of women with sufficient DNA that generated data for repeat amplification and sequencing using the PacBio single molecule real-time sequencing platform (N=60); 4 women with poor CYP2D6 metabolizer status were excluded in sensitivity analyses

[c] P-trend was calculated by modeling the time since tamoxifen initiation (T1-T3) as a continuous variable using the Wald test

[d] P-value for a Wald test of the interaction between endocrine symptom score and time since tamoxifen initiation

Supplementary Table 5. Sensitivity analyses for mean change (95% CI)[a] in breast sound speed (m/s) by endocrine symptom frequency after tamoxifen initiation

	Restricted to premenopausal women	Excluded women who discontinued tamoxifen or had undetectable levels of tamoxifen metabolites	Adjusted for antidepressant use	Adjusted for case-type	Available CYP2D6[b] status	Excluded poor CYP2D6 metabolizers[b]
Time since tamoxifen initiation						
No symptoms						
T1	-0.23 (-4.13, 3.68)	0.39 (-2.75, 3.53)	0.37 (-2.61, 3.35)	-0.01 (-2.81, 2.79)	0.70 (-2.55, 3.95)	-0.05 (-3.00, 2.90)
T2	1.42 (-4.83, 7.67)	-0.45 (-4.76, 3.85)	-0.34 (-4.54, 3.86)	-0.69 (-4.79, 3.42)	-0.32 (-4.68, 4.04)	-0.66 (-5.36, 4.03)
T3	0.83 (-4.84, 6.49)	0.84 (-3.94, 5.62)	0.53 (-3.67, 4.72)	0.14 (-3.94, 4.21)	0.41 (-4.29, 5.11)	0.25 (-4.47, 4.97)
p-trend[c]	0.17	0.47	0.91	0.91	0.96	0.98
Low/moderate						
T1	-1.28 (-3.99, 1.42)	-0.65 (-3.01, 1.71)	-0.21 (-2.66, 2.23)	-0.54 (-2.72, 1.64)	-0.35 (-2.86, 2.15)	-0.34 (-2.87, 2.19)
T2	-3.18 (-6.16, -0.20)	-1.83 (-4.37, 0.72)	-1.44 (-4.02, 1.14)	-1.78 (-4.14, 0.58)	-0.73 (-3.69, 2.22)	-0.87 (-4.35, 2.60)
T3	-4.65 (-7.82, -1.48)	-2.97 (-5.72, -0.21)	-2.11 (-4.79, 0.57)	-2.46 (-5.06, 0.15)	-2.29 (-5.14, 0.57)	-2.13 (-5.18, 0.92)
p-trend[c]	0.07	0.02	0.03	0.02	0.06	0.07
High						
T1	-0.66 (-2.95, 1.62)	-1.62 (-3.65, 0.42)	-0.98 (-3.01, 1.06)	-1.21 (-3.14, 0.71)	-1.17 (-3.50, 1.16)	-0.83 (-3.33, 1.67)
T2	-3.38 (-5.67, -1.08)	-3.31 (-5.56, -1.05)	-2.49 (-4.72, -0.26)	-2.77 (-4.93, -0.62)	-2.18 (-4.64, 0.29)	-2.12 (-4.66, 0.42)
T3	-5.25 (-8.24, -2.27)	-5.61 (-8.18, -3.05)	-4.14 (-6.49, -1.79)	-4.37 (-6.87, -1.87)	-4.77 (-7.62, -1.92)	-4.88 (-7.79, -1.97)
p-trend[c]	0.003	0.004	0.005	0.01	0.007	0.004
p-interaction[d]	0.34	0.43	0.61	0.61	0.58	0.40

Endocrine symptom frequency was assessed with a Likert scale for symptom frequency per week (vasomotor and joint pain) and per day (vasomotor only). An endocrine symptom frequency score was created by summing the values for each item and categorized into three groups defined as no symptoms (score=0), low/moderate (score=1-5), and high (score=6+)

[a] Model adjusted for age (continuous), race (white, Black/other), menopause status (premenopausal, postmenopausal), body mass index (continuous), baseline sound speed (m/s) (tertiles)

[b] Among subgroup of women with sufficient DNA that generated data for repeat amplification and sequencing using the PacBio single molecule real-time sequencing platform (N=60); 4 women with poor CYP2D6 metabolizer status were excluded in sensitivity analyses

[c] P-trend was calculated by modeling the time since tamoxifen initiation (T1-T3) as a continuous variable using the Wald test

[d] P-value for a Wald test of the interaction between endocrine symptom group and time since tamoxifen initiation

Supplementary Table 6. Distribution of tamoxifen metabolites by CYP2D6 metabolizer status[a]

CYP2D6 metabolizer status	N	(Z)-4-OH-Tamoxifen (ng/ml)[b]	(Z)-Endoxifen (ng/ml)[b]	(Z)-N-Desmethyl Tamoxifen (ng/ml)	(Z)-Tamoxifen (ng/ml)
Poor	4	1.64 (0.88-2.36)	3.02 (2.29-4.16)	256.5 (200.0-487.0)	147.0 (89.9-266.0)
Intermediate	18	1.34 (0.25-2.77)	6.03 (0.62-24.60)	212.0 (68.6-652.0)	126.0 (15.8-320.0)
Efficient	36	1.41 (0.25-6.78)	9.59 (0.25-32.4)	230.00 (2.5-280.0)	115.0 (2.5-337.0)
Ultra	0	--	--	--	--
Indeterminate	2	1.92 (0.92-2.92)	10.77 (2.83-18.70)	243.5 (228.0-259.0)	166.0 (142.0-190.0)

Values are median (minimum-maximum)
[a]Among subgroup of women with sufficient DNA that generated data for repeat amplification and sequencing using the PacBio single molecule real-time sequencing platform (N=60)
[b] 4-OH-Tamoxifen and endoxifen are synthesized via CYP2D6 with endoxifen being the most potent metabolite
